# Serious game development as a strategy for health promotion and tackling childhood obesity**^1^**

**DOI:** 10.1590/1518-8345.1015.2759

**Published:** 2016-08-15

**Authors:** Jéssica David Dias, Marcelo Shinyu Mekaro, Jennifer Kaon Cheng Lu, Joice Lee Otsuka, Luciana Mara Monti Fonseca, Silvia Helena Zem-Mascarenhas

**Affiliations:** 2 Doutoranda, Escola de Enfermagem de Ribeirão Preto, Universidade de São Paulo, Centro Colaborador da OPAS/OMS para o Desenvolvimento da Pesquisa em Enfermagem, Ribeirão Preto, SP, Brazil.; 3 Aluno do curso de graduação em Enfermagem, Departamento de Enfermagem, Universidade Federal de São Carlos, São Carlos, SP, Brazil.; 4 Aluna do curso de graduação em Imagem e Som, Departamento de Artes e Comunicação, Universidade Federal de São Carlos, São Carlos, SP, Brazil.; 5 PhD, Professor Adjunto, Departamento de Computação, Universidade Federal de São Carlos, São Carlos, SP, Brazil.; 6 PhD, Professor Associado, Escola de Enfermagem de Ribeirão Preto, Universidade de São Paulo, Centro Colaborador da OPAS/OMS para o Desenvolvimento da Pesquisa em Enfermagem, Ribeirão Preto, SP, Brazil.; 7 PhD, Professor Associado, Departamento de Enfermagem, Universidade Federal de São Carlos, São Carlos, SP, Brazil.

**Keywords:** Pediatric Nursing, Obesity, Video Games, Educational Technology

## Abstract

**Objectives:**

to develop and assess a serious game on healthy eating and physical activity to promote health and tackle childhood obesity.

**Method:**

a descriptive, applied and methodological study.For the development of the game, the following steps were taken: conceptualization, pre-production with the development of the game documentation, prototyping, production and assessment of thecomputer and health experts.

**Results:**

a prototype has been developed up to beta version. The game was positively assessed both in terms of gameplay and mechanics, and in relation to the content presented, standing out as a powerful strategy for health promotion. The information from the assessment phase contributed to the settings in the software in order to make it available in the future for the target population of this research. The greatest advantage of the proposed game is the fact that it is an open educational resource.

**Conclusions:**

the expert assessments showed that the game has great educational potential and it is considered suitable for future application to the target audience.The serious game can become a technological teaching resource available for use in schools and health facilities, and can also be reused for the production of other educational games by accessing its source code.

## Introduction

The global and Brazilian panoramas on obesity have emerged as a new challenge for public health because its incidence and prevalence has increased alarmingly in the last 30 years^1^. In 2010, it was estimated that 43 million children in the world are already considered overweight and suffering from obesity^2^. 

In order to minimize the problem of overweight and obesity, health policies in Brazil and in the world have established a series of interdisciplinary and multisectoral objectives with the aim of promoting health^3^ ^-^ ^4^.It is noticed the need to use different strategies to achieve health education, especially those focused on children, such as games^5^. 

An educational approach based on electronic games may include entertaining features and specific content to promote the child's learning process^6^. The use of games and fun activities may be a useful tool and well accepted by children to achievethe health education goals.

It is highlighted in this scenario, the importance of integrating the serious game in the educational context. A serious game is a game in which education, in its various forms, is the main objective^7^. These games promote learning and behavioral changes^6^.

In studies published in the scientific literature, serious games were used as educational tools in an innovative way. These games have been used successfully in the area of health, which helps in the treatment of children and adults with chronic diseases (such as diabetes, asthma and cancer) or under psychotherapeutic treatment^8^ ^-^ ^9^.

In this context, the aim of this study was to develop and assess a serious game about healthy eating and physical activity to promote health and tackle childhood obesity.

## Method

Descriptive, applied and methodological study, with the purpose to develop and assess a serious game with computer and health experts. This type of study involves the development, validation and assessment of technological tools and research methods^10^. The studies carried out by Schell^11^ and Novak^12^ supported the methodological design and construction of the required documents.

As for the literature of game design, a game can be understood as the composition of four elements, considered as the elemental tetrad: mechanics, aesthetic, narrative and technology. Mechanics is the operation of the game, narrative is the sequence of events during the course of the story to be told, esthetics is composed by audiovisual components and emotions, while technology is represented by the media^11^. As for the methodological approach, a game can be developed from five stages: concept, pre-production, prototype, production and post-production^12^.

To develop the serious game proposed in this study, the following steps were taken: conceptualization, pre-production with the development of the *Game Design Document* (GDD), prototyping, production and assessment of the experts.

In addition, studies and researches in the areas of design and assessment of educational games were performed by means of weekly meetings with participation of the students and teachers responsible for the project. It was also carried out a survey on the demands of content with nutrition professionals and the use of iterative cycles, that is, cycles in which it is possible to return to any previous stage whenever it is necessary in order to improve the development of the game. Subsequently, the design and construction of the game were performed based on the identified needs. 

The iterative cycle of the game at issue started with the design and development of the initial idea, which was presented to the team and after several suggestions, the production of the low-fidelity prototype began. Subsequently, after the survey and resolution of the problems of this stage, a phase of high-fidelity prototyping started. At the end of this phase, content and computer experts were invited to assess the latest version. 

The assessments were carried out by computer experts regarding the usability and procedure issues, as well as assessments of educational content by health experts, considered as experts on the subject. In compliance with the ethical and scientific rigor, the study received a favorable opinion from the Ethics Committee on Human Research, under protocol number 346.216/2013. Data were collected in the second half of 2014, only after the participants have accepted and signed the Informed Consent Form (IC).

In the assessment of the first playable version of the serious game, semi-structured questionnaires based on the EGameFlow method for assessing educational games were used^13^, adaptated for educational games and derived from the GameFlow method, which focuses on the assessment of games. This questionnaire was divided into seven categories (Concentration, Challenges, Autonomy, Clarity of Objectives, Feedback, Immersion and Improvement of Knowledge). The items of the instrument ranged from 1 to 7, where 1 isconsidered as "weak" and 7 as "strong". In this study, the criteria with the final average equal to or less than 6 have been considered as items that must be rethought in order to improve the final version of the game^13^.

The data collection instrument used in the assessment of the experts was initially translated, adapted and previously applied for the assessment of educational games, by the Laboratory of Learning Objects (LOA)^14^, an interdisciplinary environment for the study and research of new methodologies and technologies for the development of games and Open Educational Resources (OER), and permission from the authors was required for the use of the instrument.

For the assessment of the game proposed in this study, 10 assessors were selected: six content experts with a degree in health (nutrition and nursing) and at least one year training andmaster's degree, as well as experience with the theme health technologies for assessment of content, and four computer experts with a degree (computer science and systems analysis) and at least one year training andmaster's dregree in the areas of software development and/or digital learning games, to assess the gameplay,mechanics and game interface.

The assessors were chosen according to an adaptation of the Fehring Model^15^, for the selectionof the specialists. They were screened through a search at Lattes Curriculum and it was requested to the first experts selected to indicate other assessors with the same characteristics. An email was sent to 12 experts to invite them to participate in the survey and clarify the objectives and methodology used, however, two refused to participate because they were on vacations or maternity leave. After the acceptance of the 10 assessors, it was arranged a time and place for onsite assessment of the game, followed by the application of the EGameFlow questionnaire. 

## Results

The serious game was named as DigesTower and developed by four undergraduate students (Computer Science, Nursing, Music and Image and Sound) and a Master's student in Nursing, under the coordination of Professors in the areas of Computer Science and Nursing. Since this is an interdisciplinary group, the game was developed according to a collaborative and balanced approach. Illustrators and programmers were engaged in drawing up a fun and functional mechanism, while the teachers and students responsable for the content to be inserted in the game discussed and developed a meaningful and educational learning system, in order to overcome the major challenge of the project: prevent that the educational goals were overshadowed by the narrative and other elements.

To assist in the development of the game, the team relied on a set of activities such as brainstormings; literature searches; development of script and documentation; development of audiovisual components; coding and then, tests and assessments.Some of the features of the DigesTower game are described as follows*.*

*Game Summary:* DigesTower has the human digestive system as background and is classified as a tower defense type of game. Tower defense games can be classified as a subtype of strategy game, and as their name suggests, they are focused on the defense of an element of the game^16^.The game has school children as its target audience.The main character is called Elise. She gets hungry and goes to the refrigerator to choose something to eat and the game starts. The food enters the body at regular intervals and the digestive enzymes digest each feed in its proper organ. The game has three phases and seven levels. When the game starts, the food is displayed according to its class (carbohydrates, proteins and fats) and the entire digestive tract is illustrated with its principal organs. There are also moments of explanations on the organs and on the specific digestion of each food class. At the end of each stage, the players receive feedback on the health of the character and their progress in the game.

*Educational objectives:* the following educational objectives were chosen: (1) understand the importance of healthy eating; (2) understand the importance of physical activity; (3) understand and distinguish in which organ each class of food is digested; (4) understand that excessive consumption of fat is harmful; (5) understand the organs responsible for the digestion and enzyme action through the digestive system. Learning takes place gradually, beginning with basic items such as the presentation of foods composed of carbohydrates and presentation of the region of the mouth, ending at a more advanced level, with exposure of other organs belonging to the digestive system.

*Narrative:* the background is composed of the human digestive system.The protagonist is Elise, a school-age childand there are foods and fat as obstacles. The game initially presents an animation of Elise hungry and opening the refrigerator and at the end it shows a happy child brushing the teeth.

**Figure 1:**
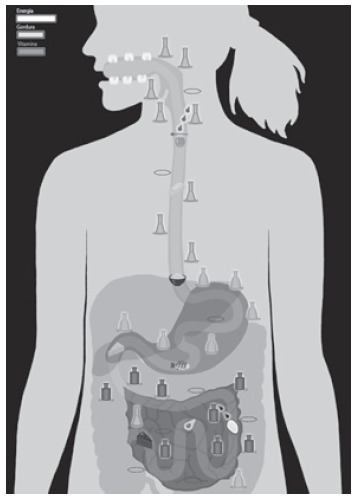
Game map and pathwayof food

**Figure 2:**
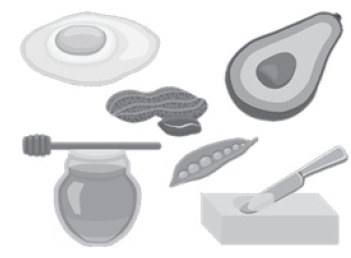
Examples of foodof the game

**Figure 3:**
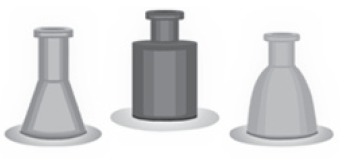
Examples of digestive enzymes ("towers")

*Esthetics:* the game features a playful tone, but educational and realistic (since it considers the proportion of the digestive organs) and is composed of cartoon-like drawings*.* However, foods are not shown in realistic proportions, aiming at their better identification in the game map (Figures 1 and 2). The towers represent the digestive enzymes and are also not shown in the reliable format of the actual enzymes (Figure 3).

*Mechanics:* the game map consists of organs of the digestive system (mouth, esophagus, stomach and intestines). The towers are represented by digestive enzymes, food is divided by food groups into carbohydrates, proteins and lipids. Fat is represented in some food of the lipid class that release low density fats and are accumulated between the organs, preventing the passage of the next food.There are four types of bars: Health; Energy, Vitamin; Fat.Finally, the specials, which are resources available to help the player to digest food faster and that are activated when the vitamin bar is filled.The DigesTower has the same rules of Tower Defense games. In these games, the "enemies" appear grouped and move towards the end of the stage in which the main target is. The player has a pre-defined space and uses a defensive strategy^16^.In the case of DigesTower, the food comes in groups of each food class and move through the digestive tract.

During the game, it is possible to choose the appropriate locations of each tower, the special items and the best time to run the special elements, with the aim of favoring the digestion of food. For example, when there are many foods of the carbohydrate class, the player can use the special device "saliva rain" in order to speed up the digestion.If a large amount of food reaches the end of the level without being completely digested, the "indigestion bar" is filled up and the player loses the game, however, if the towers are able to digest the food and the bar is not completely filled, the player wins.

*Technology:* Unity 3D technology was used, which contains several features that facilitate and help in designing games in two or three dimensions, providing greater practicality in programming. The game was developed for computers and will be subsequently adapted for tablets. 

*Prototyping and production:* the implementation of the game started after planning and detailed study on how the basic elements would constitute the gameplay. The development and codification of the game, as well as the content of the productions, visual elements and planning phases occurred jointly with the educational goals, through a process of intense reflection and updating of the design. At the end of a year of work, the group was able to produce the first complete version of the serious game. The game was developed up to the *beta* version, that is, all the game components were incorporated into the game and the production process was concluded. The *beta* phase aims to stabilize the game and eliminate the greatest number of defects before making the product available to the public^12^.

The game has three complete phases (consisting of seven levels of difficulty), scoring framework for each food, bonus of vitamins, specials, narrative and game explanation, screens related to educational goals, as well as basic elements of the game such as menus, tracks and sound effects, home screen and stages of tutorials. The *beta* version of DigesTower is available for access on the website of the LOA and can be accessed and used by anyone. The game features the Creative Commons license and is available as an open educational resource. The source code is shared with the free software community through Git Hub system.

*Expert assessments:* The first functional version (*Alpha*) of the game was submitted toa more accurate assessment by health and computer experts, that is, a version that can be played in full, but still contains bugs and undefined elements^12^.

The computing assessors had a dregree in Computer Science and Systems Analysis. Two participants were male and two females and the age ranged from 22 to 30 years. The health assessors had a degree in Nutrition and Nursing. All six assessors were female and aged between 23 and 37 years old.

The expert assessments were performed using the EgameFlow questionnaire (Table 1). Since this is a fairly complete questionnaire and has potential to cover both areas of knowledge (Computer Science and Health), although with different approaches, there was no damage to the assessment of the game at issue.

**Table1:** Assessment averaging of the EgameFlow instrument. São Carlos, SP, Brazil, 2014

Item	Criteria	Mean
Concentration	1 The game grabs my attention?	6.5
	2 Most activities are related to the learning task?	6.4
	3 I am not distracted fromthe tasks that I should concentrate on?	6.3
	4 I am not burdened with tasks that seem unimportant?	6.3
Challenges	5 I enjoy the game without feeling bored or anxious?	6.2
	6 The challenge is adequate?	5.3
	7 There are "hints" that help me in the task?	5.4
	8 The game provides information, on demand, to help me in the task?	5.3
	9 My skills improve as the game progresses?	6.3
	10 I am encoraged by the improvement of my skills?	6.3
	11 The challenges increase as my skillsimproved?	6.1
	12 The game provides new challenges with an appropriate pacing?	6.0
	13 The game providesdifferent levels of challenges to suit different players?	5.8
Autonomy	14 I feela sense of control the menu?	6.3
	15 The game does not allow me to make serious errors that prevent me to progress in the game?	5.4
	16 The game supports my recovery fromerrors?	5.7
	17 I feel I can use other strategies?	6.3
	18 I know the next step in the game?	6.1
	19 I feel a sense of control over the game?	6.1
	20 I feel that my actions have a significant impact over the game?	6.2
Clarity of Objectives	21 Overall game goals are presented in the beginning of the game?	6.3
	22 Intermediate goals are presented in each stage or level?	4.3
	23 I understand the learning goals throughout the game?	6.3
Feedback	24 I receive feedback on my progress in the game?	5.8
	25 I receive immediate feedback on my actions?	6.0
	26 I receive information on my success or failures of intermediate goals?	6.2
	27 I receive information on my status, such as level or score?	5.9
Immersion	28 I forget about time while playing the game?	6.4
	29 I forget about my surroundings while playing the game?	6.6
	30 I forget the problems of day-to-day while I play?	6.3
	31 I become involved in the game?	6.6
Improvement knowlegde	32 The game improves my knowledge?	6.6
	33 I catch the basic ideas of the presented content?	6.5
	34 I try to apply the knowledge in the game?	6.3
	35 I try to know more about the presented content?	6.4

Small differences were observed in the focus of the assessments of the experts, and those concerning the area of health were more focused on the content of the screens and almanacs of the game, as well as on the illustration of food. The computing assessments were more focused on the mechanism, interface and gameplay, although all these areas are encompassed by the instrument. According to the answers of the first category, the assessors pointed out that they remained very concentrated on the game. For them, the game's activities are consistent, direct and easy, thus favouring the concentration.

The experts felt challenged during the game and assessed that it has fulfilled the requirements with regard to the category "Challenge". The score generated for the item 6 is justified because this is an *alpha* version of the game and, therefore, there were still problems regarding the balance and adaptation to the different levels of difficulty at the time of the tests, which were already refined and suitable for the release of the *beta*version of the game. 

Regarding the items 7 and 8, which refer to the help tips on the tasks of the game, it is considered that they have been revised for the *beta* version, however, the game was still under development and the screens with help hints had not been added to the prototype until the moment of the test by the assessors.

Based on the answers given in the third category, it was found that the evaluators had good autonomy. The items 15 and 16 were subjected to error recovery during the game and their scores are justified because it was a version of the gamestill under development. These assessors pointed out some errors during their tests that had not been found by the team, and therefore their contributions and the programming errors raised for correction and improvement of the game until the final version were of great importance.

It was also noted that the experts were able to identify positive and negative feedback according to their actions during the game and it is believed that the game has fulfilled the requirements with regard to the category "Feedback". It was suggested a more evident display of the basic information such as the scoring, the values of the towers and level in the game in the graphical interface, justifying the items with scores below 6. 

Based on the responses of the items 32 to 35, it was observed that the assessors were immersed in the game, forgetting about the time, the surrounding environment and day to day problems. In addition, in the open answers, the experts saidthey had improved their knowledge with the game and assessed it positively, considering it as innovative and powerful for future use with the target audience.

## Discussion

In the health sphere, the serious games have been used in different contexts, exploring the immersion of players to achieve educational goals^17^ ^-^ ^19^. At the end of this study, it was noted that the objectives have been achieved and it was possible to analyze the repercussions of the serious game as an educational technology, along with the experts. It also was found that the game has an innovative and educational potential according to the assessors.

In sum, it is emphasized the need for the development and evaluation of new technologies in the health area, particularly the educational technologies in order to capture the attention of the audience in a different way, especially if that audience belongs to the present generation, known as digital generation, already used to using the cyberspace and that has preference for new technologies compared to traditional strategies of health promotion^12^. 

The serious game DigesTower was proposed and developed in order to accomplish this purpose. It was sought to offer a quality game for children of school age so that they could use it as a fun and engaging way to learn about human digestion, healthy eating and physical activity, thus promoting health. 

The game stands out by combining the healthy eating theme with the mechanics of the Tower Defense game. In addition, in order to stimulate learning of the player and encourage behavioral changes, there was great concern in combining the educational objectives with the gameplay, and therefore the game had the participation of educators and health professionals from its design to its implementation.

One of the biggest advantages of the proposed game is that it is a free and open educational resource. The DigesTower can be considered as a resource for technological education with free access for use in schools and health facilities, and can be reused for the production of other games in the area through access to its source code.

It may be noted that the identification of the player with the serious game, through immersion, favors the entertainment and development ofthe teaching and learning processes^20^. In this regard, it was sought to meet these demands during the DigesTower game design. During the development process, it was sought to combine aspects of learning with the interface, audio and esthetics, providing a greater immersion and adhesion of the player, in order to achieve the proposed educational objectives.

Furthermore, the serious game has proven its importance and suitability through the assessment by the experts in the fields of health and computing, which reinforces the importance of the validation stage and corroborates the findings of other studies on the validation of serious games in the area of health^21^ ^-^ ^22^. 

The main deficiency found has been the lack of opportunity to test the game with the target audience to achieve more effective results and the possibility of validation of the game in practice as an intervention. Thus, it is intended to combine the use of the serious game with strategies for the prevention and treatment of childhood obesity, since studies show that there has been a meaningful return on the use of this type of tool with this public^19^ ^,^ ^23^. 

Thus, it is intended to improve the game through refinements and to continue the tests with the target audience in future studies, in order to subsequently insert it into educational programs that address obesity. 

## Conclusion

In this study it was possible to identify the stages of the development and evaluation process of the game DigesTower. The game is freely available as an open educational tool, thus benefiting the academic community and society in general. 

The combination of computing resources with education represents another way to participate in tackling childhood obesity. Thus, the proposal to develop a serious game was very relevant. The game can be considered an innovative strategy in order to compose as an additional intervention to tackle childhood obesity and may serve as basis for future studies on the same theme, representing new health promotion strategies.

Through the assessments of the computing and health experts, it was possible to notice that the game has great potential as an educational tool, considering that it was well assessed both regarding its mechanics and gameplay, and regarding the educational content since it was considered appropriate for future application in the target audience. 
